# Graphene Quantum Dots-ZnS Nanocomposites with Improved Photoelectric Performances

**DOI:** 10.3390/ma11040512

**Published:** 2018-03-28

**Authors:** Zheng Zhang, Chengyi Fang, Xin Bing, Yun Lei

**Affiliations:** School of Resources and Environmental Engineering, Wuhan University of Technology, Wuhan 430070, China; 219915@whut.edu.cn (Z.Z.); fcy1255411153@163.com (C.F.); 15927301445@163.com (X.B.)

**Keywords:** GQDs, ZnS-GQDs composites, photocurrent responses, electrochemical impedance spectra

## Abstract

ZnS-graphene quantum dot (GQDs) composites were synthesized by a simple solvothermal method, in which GQDs were prepared by a hydrothermal cutting process. The products were characterized by transmission electron microscopy, atomic force microscopy, X-ray diffraction and ultraviolet-visible absorption spectroscopy. The results show that GQDs were obtained by size tailoring of 1–4 graphene layers and combined with cubic ZnS nanoparticles to form ZnS-GQDs composites. The photocurrent and electrochemical behavior of the products were evaluated by transient photocurrent responses and electrochemical impedance spectra. The photocurrent density of ZnS-GQDs achieves the value of 2.32 × 10^−5^ A/cm^2^, which is 2.4-times as high as that of ZnS-graphene. GQDs serve as an electrical conducting material, which decreases the conductive path and accelerates the electron transfer. The charge-transfer resistance of ZnS-GQDs is much lower than that of ZnS-graphene and pure ZnS due to the effective electron separation and transfer ability upon the incorporation of GQDs.

## 1. Introduction

As an important direct-band semiconductor, zinc sulfide has attracted much attention in photoelectric characteristics due to its rapid generation of electron-hole pairs by photoexcitation [1,2,3]. However, photo-generated electrons from ZnS tend to recombine with holes, which results in an obvious decrease in the separation and transfer of photoelectrons. To overcome the problem, it is a good way to combine ZnS nanostructures with different semiconductors and carbon materials [4,5,6,7,8]. Pal et al. investigated the photocurrent generation and photocatalytic activity of one-pot solvothermally-synthesized reduced graphene oxide (RGO)-ZnS composites [9]. Wang et al. achieved graphene/ZnS nanowire film hybrid-based photodetector arrays for high-performance image sensors [10]. Jia et al. employed a low-temperature solid-state method to obtain graphene/ZnS nanocomposites with enhanced photocatalytic activity [11]. Liu et al. prepared ZnS-graphene hybrid nanomaterials with enhanced electrochemical activity and stability [12]. The performances of ZnS-graphene were improved by combining ZnS with graphene sheets, but the sheets have some shortcomings like easy aggregation and poor dispersion in solvents. Moreover, graphene-based materials are generally micrometer-scale graphene sheets, which further limits their application in nanodevices.

Different from graphene sheets in a two-dimensional structure, GQDs represent a kind of zero-dimensional carbon nanoparticle obtained by size tailoring of single or few-layered graphene. Several approaches have been applied to prepare GQDs including hydrothermal or solvothermal cutting [13,14], electrochemical synthesis [15], nanolithography [16], the microwave-assisted method [17] and ultrasonic shearing [18]. Owing to the excellent optical, electrical and electrochemical properties, GQDs are regarded as promising candidates for semiconductor hybrids. Hsu et al. demonstrated that CdSe/GQDs exhibited higher photocurrent generation as compared to pure CdSe and CdSe/RGO [19]. Chu et al. fabricated SnO_2_/GQDs nanocomposites via the solvothermal method and investigated the effect of the GQDs content on the gas-sensing responses and selectivity [20]. Lei et al. fabricated CdS/GQDs by a two-step hydrothermal deposition method and achieved a highly efficient photocatalytic hydrogen evolution [21]. Wang et al. fabricated GQDs-TiO_2_ by a simple physical adsorption method. The photocurrent enhancement was ascribed to the synergistic amplification of TiO_2_ and GQDs [22]. Jang et al. developed a two-step route to synthesize GQDs-decorated ZnS nanobelts that exhibit enhanced photocatalytic performance [23]. GQDs act as a conductive path for electron separation and transfer. Therefore, the combination of semiconductors and GQDs seems to improve charge separation by hindering charge recombination, thus enhancing photoelectrochemistry properties. The reported works are based on a two-step route in which semiconductor materials were required to prepare in advance. The two-step approach is limited by relatively complicated process and a long reaction time. Therefore, it is more economical and reasonable to synthesize composites through a simple and straightforward method.

Herein, ZnS-GQDs composites were prepared by a one-step solvothermal synthesis, in which GQDs were synthesized by a hydrothermal cutting method. The products were characterized by transmission electron microscopy, X-ray diffraction and ultraviolet-visible absorption spectroscopy. Further photoelectric and electrochemical performances were analyzed by transient photocurrent responses and electrochemical impedance spectroscopy.

## 2. Materials and Methods

### 2.1. Materials

Graphite powders (99%), concentrated sulfuric acid (H_2_SO_4_), sodium nitrate (NaNO_3_), potassium permanganate (KMnO_4_), hydrogen peroxide (H_2_O_2_), anhydrous ethanol (CH_3_CH_2_OH), zinc acetate dihydrate (Zn(CH_3_COO)_2_·2H_2_O), thiourea (SC(NH_2_)_2_), sodium hydroxide (NaOH), concentrated nitric acid (HNO_3_), ethylene glycol (CH_2_OHCH_2_OH) and polyethylene glycol (PEG) were commercially available products and used without further purification.

### 2.2. Preparation of Graphite Oxide and Graphene

Graphite oxide was prepared by a Hummers process, including three stages of low, medium and high temperature. At low temperature, graphite powders (5 g), potassium permanganate (15 g) and sodium nitrate (2.5 g) were mixed with concentrated sulfuric acid (115 mL) at 4–6 °C for 90 min. Then, the mixture was heated at 38 °C for 30 min for further oxidation of the graphite intercalation compound. In the high temperature process, distilled water (200 mL) and hydrogen peroxide (30 mL) were added into the mixture, and the mixture was heated at 90–100 °C for 20 min. The produced graphite oxide was rinsed with distilled water and dried in a vacuum oven (TAISITE Instrument, Tianjin, China) at 45 °C. Graphite oxide was treated with thermal annealing at 300 °C for 30 min to obtain graphene in nitrogen flow.

### 2.3. Preparation of GQDs

GQDs were prepared with a two-step process. Firstly, graphene (100 mg) was mixed with concentrated nitric acid (60 mL) and concentrated sulfuric acid (20 mL). After ultrasonic treatment for 10 h, the mixture was rinsed with distilled water to neutral and dried in a vacuum oven at 45 °C. In the second step, a dry sample (50 mg) was dispersed in 100 mL distilled water and sonicated for 1 h. The solution was adjusted to the pH value of 8 with the help of sodium hydroxide. After the mixture was transferred to an autoclaved in a Teflon-lined stainless steel vessel, the reaction system was heated at 200 °C for 10 h. The mixture was filtered by a microporous membrane (0.22 μm), and the filtrate was dialyzed (3500 Da) for 24 h to obtain GQDs.

### 2.4. Synthesis of ZnS-GQDs Composites

ZnS-GQDs composites were prepared by a one-step solvothermal process using zinc acetate dihydrate as Zn precursors, thiourea as S precursors and ethylene glycol as solvents. At first, zinc acetate dihydrate (0.15 mmol) and GQDs were dissolved in 30 mL ethylene glycol. Then, thiourea (0.15 mmol) was added into the above solution, and the mixture was stirred for 20 min. Lastly, the mixture was autoclaved in a Teflon-lined stainless steel vessel at 200 °C for 12 h.

### 2.5. Characterization

The morphology of ZnS-GQDs composites was observed on a JEM-2100F transmission electron microscope (TEM, JEOL, Tokyo, Japan) and a Bruker MultiMode 8 atomic force microscope (AFM, Bruker, Billerica, MA, USA). X-ray diffraction (XRD, Bruker, Billerica, MA, USA) was performed on a D8 Advance diffractometer using the monochromatized X-ray beam from Cu Kα radiation. UV-Vis adsorption spectra were obtained using a UV-Vis spectrophotometer (UV5500, Shimadzu, Kyoto, Japan). Transient photocurrent responses (I-T) and electrochemical impedance spectroscopy (EIS) were measured at a CHI650E electrochemical work station (Chenhua Instruments, Shanghai, China). A glassy carbon electrode coated with samples, a platinum foil and a standard calomel electrode served as the working electrode, the counter-electrode electrode and the reference electrode, respectively.

## 3. Results and Discussion

### 3.1. XRD

**Figure 1:**
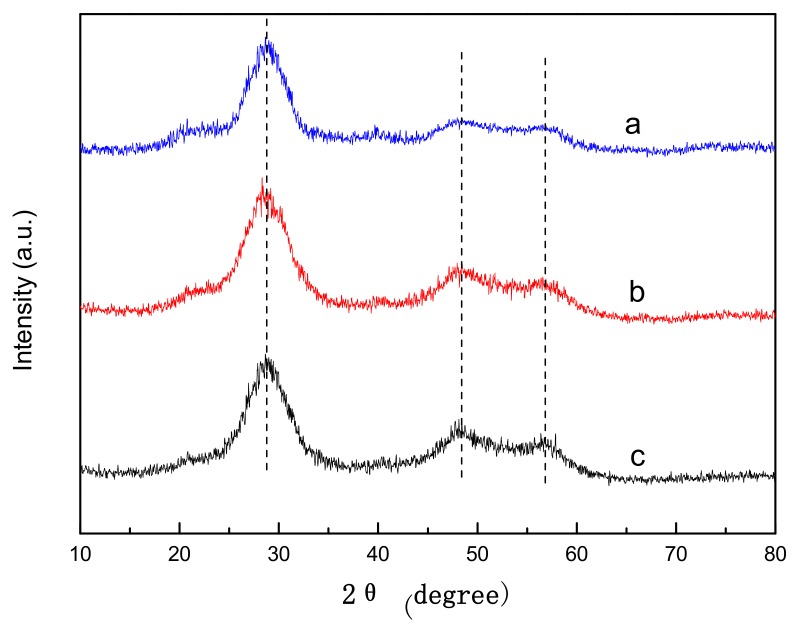
XRD patterns of ZnS-GQDs (a), ZnS-graphene (b) and pure ZnS (c).

The X-ray diffraction pattern of ZnS-GQDs is shown in Figure 1a. Three obvious diffraction peaks at 28.73°, 48.39° and 56.81° could be indexed as the (111), (220) and (311) planes of the cubic ZnS corresponding to the standard card (JCPDS 77-2100). The crystallinity of ZnS-GQDs (Figure 1a) is similar to that of ZnS-graphene (Figure 1b) and pure ZnS (Figure 1c). The result indicates that ZnS can nucleate and grow in the presence of GQDs or graphene.

### 3.2. Morphological Characterization

**Figure 2:**
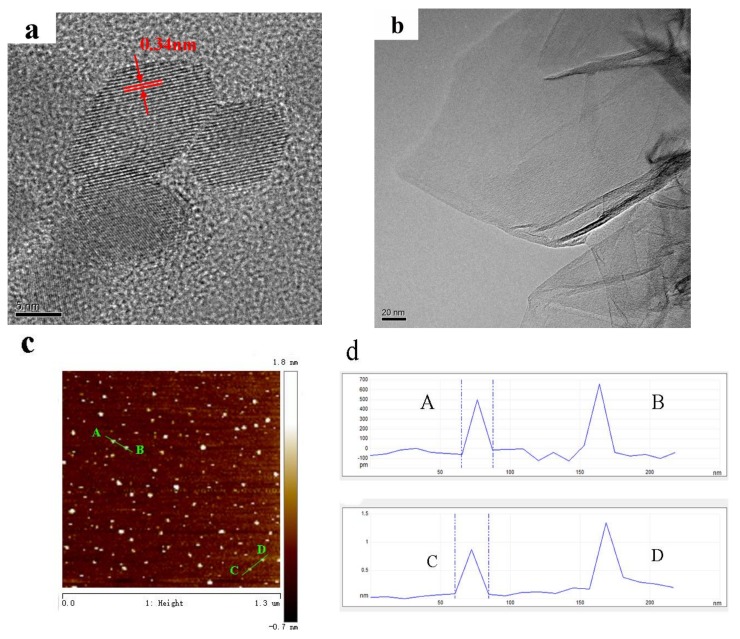
TEM image of graphene (**a**); HRTEM image of GQDs (**b**); AFM graph (**c**) of GQDs and height profile graph (**d**) of GQDs.

The morphologies of GQDs and graphene were characterized by TEM and AFM analysis. Figure 2a shows the HRTEM images of GQDs. The lateral size of GQDs is about 10 nm with the lattice spacing of 0.34 nm. When zero-dimensional GQDs were extended to two-dimensional graphene sheets, the morphology is present in Figure 2b. The graphene sheets have a corrugated structure with micrometer-long wrinkles. AFM was used to analyze the size and height profile of the GQDs. As shown in Figure 2c,d, GQDs are uniformly dispersed with a few graphene layers. The average height of GQDs is 0.5–1.5 nm, indicating that GQDs are roughly 1–4 layers.

**Figure 3:**
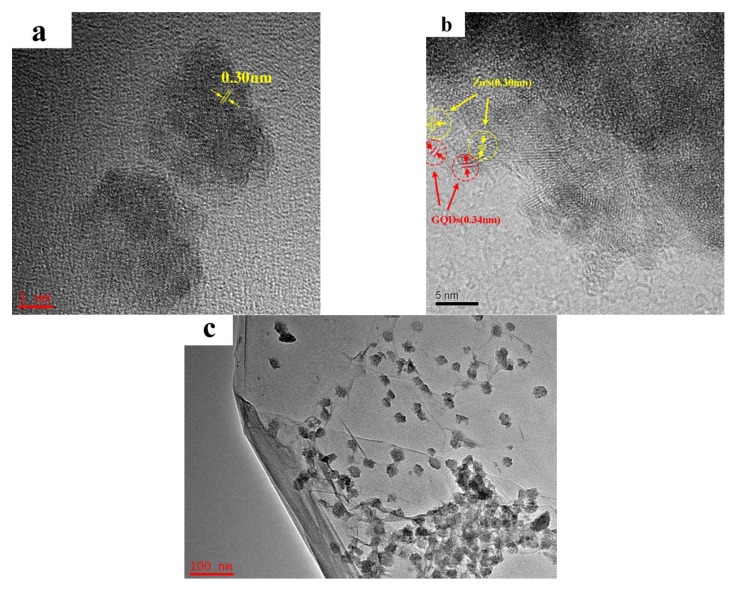
TEM images of ZnS-graphene (**a**) and ZnS-GQDs (**b**,**c**) composites.

ZnS-GQDs composites were characterized by TEM analysis as shown in Figure 3a,b. It can be observed that ZnS-GQDs composites have a spherical-like morphology. The lattice spacing of 0.30 nm and 0.34 nm corresponds to the (111) plane of ZnS and the (002) plane of GQDs, respectively. The TEM image of ZnS-graphene is shown in Figure 3c. ZnS nanoparticles are distributed on the base plane of graphene sheets with a particle size of about 30 nm.

### 3.3. UV-Vis Absorption Spectra

**Figure 4:**
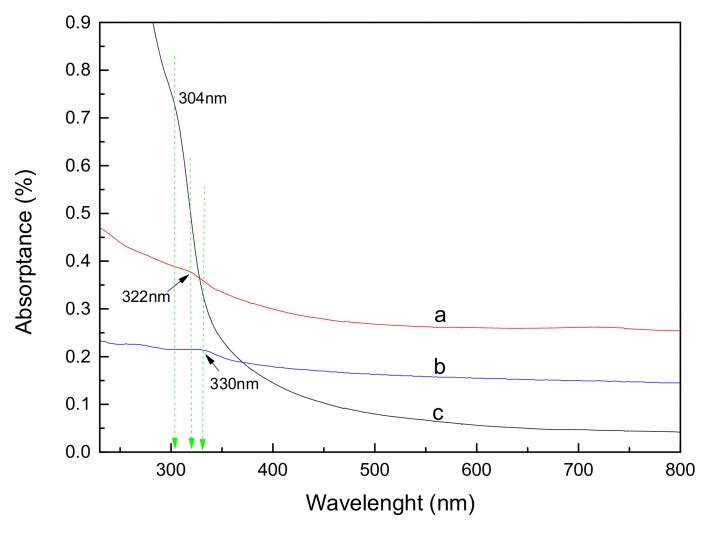
UV-Vis absorption spectra of ZnS-GQDs composites (a), ZnS-graphene composites (b) and pure ZnS (c).

Figure 4 shows the absorption spectra of ZnS-GQDs, ZnS-graphene and pure ZnS. ZnS-GQDs present a typical absorption peak at about 270 nm, which is ascribed to the excitation of the π-plasmon of the graphitic structure. Similarly, an absorption feature at about 270 nm corresponds to the characteristic peak of graphene in Figure 4b. The results show that the oxygen-containing groups have been removed in the structures of graphene and GQDs composites. A shoulder at 304 nm corresponds to the absorption peak of ZnS in Figure 4c. Compared to the pure ZnS, the absorption peaks of ZnS-GQDs and ZnS-graphene shift to 322 nm and 330 nm, respectively. The red-shift might be ascribed to the interaction between GQDs/graphene and ZnS [24].

### 3.4. Transient Photocurrent Responses

**Figure 5:**
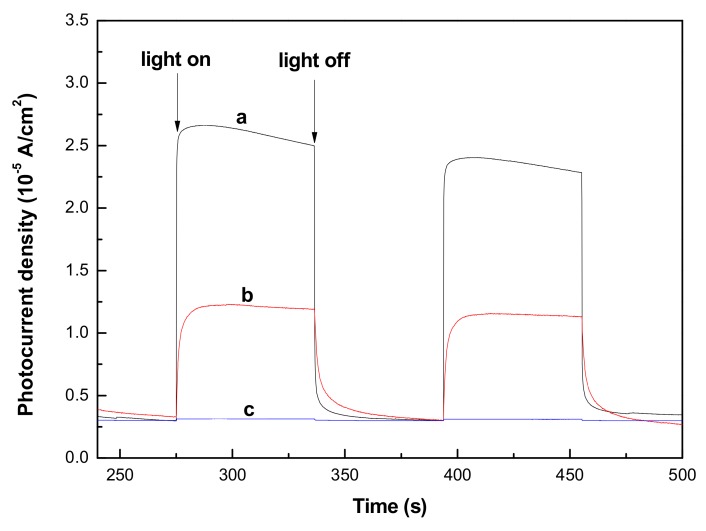
Transient photocurrent responses of ZnS-GQDs (a), ZnS-graphene (b) and pure ZnS (c).

Figure 5 presents a comparison of transient photocurrent-time curves for ZnS-GQDs, ZnS-graphene and pure ZnS under intermittent light illumination. When the light was illuminated on the surface of samples, a part of the photo-generated electrons was transported to form a stable photocurrent, but most of the photo-generated electron-hole pairs were easy to recombine. The electron capture and separation abilities of GQDs or graphene promote a higher photocurrent response of ZnS-GQDs (Figure 5a) and ZnS-graphene (Figure 5b) as compared to that of pure ZnS (Figure 5c). As shown in Table 1, the photocurrent density of ZnS-GQDs achieves the value of 2.32 × 10^−5^ A/cm^2^, which is 2.4-times as high as that of ZnS-graphene. The photocurrent improvement could be ascribed to the reason that the nano-sized GQDs tend to reduce the conduction path of photoelectrons and promote a more efficient separation of photoexcited carriers from ZnS-GQDs.

### 3.5. Electrochemical Impedance Spectroscopy

**Figure 6:**
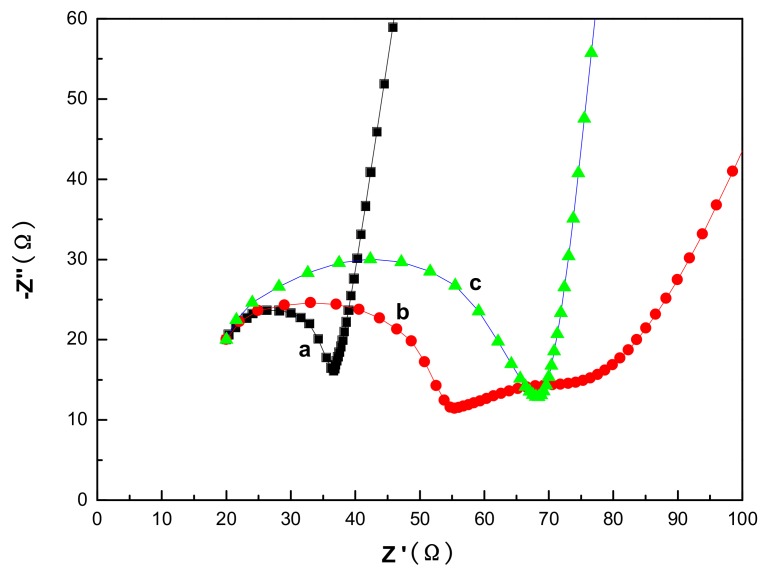
EIS of ZnS-GQDs (a), ZnS-graphene (b) and pure ZnS (c).

Electrochemical impedance spectroscopy (EIS) was performed on a three-electrode system to investigate the electron transfer process at an interface modified electrode. Figure 6 shows the EIS Nyquist plots of ZnS-GQDs, ZnS-graphene and pure ZnS in the frequency range of 1000 kHz–0.1 Hz. The impedance plots consist of a semicircle arc followed by a straight line. The semicircle arc is associated with the charge-transfer resistance between the electrode and the electrolyte solution. Compared to pure ZnS, the EIS Nyquist plots of ZnS-GQDs and ZnS-graphene have smaller semicircles due to the existence of GQDs and graphene. The semicircle diameter of ZnS-GQDs is much smaller than that of ZnS-graphene. The result indicates that the incorporation of GQDs decreases the charge-transfer distance and improves the electrical conductivity and mobility of ZnS-GQDs.

## 4. Conclusions

GQDs were prepared by a hydrothermal cutting method, and ZnS-GQDs composites were synthesized by a simple solvothermal process. The results show that ZnS-GQDs have a spherical-like morphology with the lattice spacing of 0.30 nm and 0.34 nm corresponding to the (111) plane of ZnS and the (002) plane of GQDs, respectively. The absorption peaks of ZnS-GQDs and ZnS-graphene present a red-shift to 322 nm and 330 nm due to the interaction between GQDs/graphene and ZnS. The photocurrent density of ZnS-GQDs reaches the value of 2.32 × 10^−5^ A/cm^2^, which is 2.4-times as high as that of ZnS-graphene. The enhanced photocurrent activity can be ascribed to the effective electron separation and transfer ability upon the incorporation of GQDs, which greatly decreases the charge-transfer distance and improves the electrical conductivity and mobility of ZnS-GQDs, as well as reducing the charge-transfer resistance.

## Figures and Tables

**Table 1:** The photocurrent density value of ZnS-GQDs, ZnS-graphene and pure ZnS.

Sample	ZnS-GQDs	ZnS-Graphene	ZnS
the photocurrent density (A/cm^2^)	2.32 × 10^−5^	0.97 × 10^−5^	0.13 × 10^−6^
